# Moral harassment and mental health in medical residents: a longitudinal study

**DOI:** 10.47626/1516-4446-2024-3579

**Published:** 2025-01-22

**Authors:** Ana Bresser Tokeshi, Renato Antunes dos Santos, Luiz Antonio Nogueira-Martins, Maria do Patrocinio Tenório Nunes, Thiago Marques Fidalgo

**Affiliations:** 1 Departamento de Psiquiatria, Escola Paulista de Medicina, Universidade Federal de São Paulo, São Paulo, SP, Brazil; 2 Department of Psychiatry, University of Toronto, Toronto, ON, Canada; 3 Department of Psychiatry, McMaster University, Hamilton, ON, Canada; 4 Departamento de Medicina Interna, Faculdade de Medicina, Universidade de São Paulo, São Paulo, SP, Brazil

**Keywords:** Hidden curriculum, moral harassment, medical education, residency, internship

## Abstract

**Objective:**

This study investigated whether moral harassment contributes to anxiety, depression, and burnout among medical residents.

**Methods:**

This three-stage longitudinal study involved 218 1st-year residents, of whom 76 (34.9%) participated in all stages. The questionnaire covered demographics, mental health (using the Patient Health Questionnaire-4), burnout (using the Maslach Burnout Inventory Human Services Survey), and harassment experiences. Mental health outcomes and harassment were analyzed using logistic regression.

**Results:**

Anxiety and depression scores varied significantly, including a notable decrease in the personal accomplishment dimension of burnout. The prevalence of harassment was above 90%, and most victims were disturbed by the harassment they suffered. While a direct correlation between harassment victimization and reduced mental health was not found, seeking help exacerbated suffering, and depression and emotional exhaustion increased less among surgical residents.

**Conclusion:**

To the extent of our knowledge, this is the first longitudinal study on mental health and harassment among medical residents. The mental suffering experienced after taking action against harassment suggests that safe environments for addressing these issues are lacking in medical residencies. Further studies concerning surgical residents could shed light on their lower levels of suffering. Institutional changes are needed to support victims and create a healthy environment.

## Introduction

Medical education is a complex subject. While medical knowledge grows exponentially, it is a continuous challenge to prepare young doctors for this ever-transforming world. However, as the medical community rushes towards rational and scientifically based conduct, mental health issues among medical students, residents, and professionals are increasing. The medical community has a higher prevalence of anxiety, depression, burnout, and suicide than the general population, and medical residents also follow this trend.^1^-^4^ For this reason, the stress experienced by residents has been studied in many countries. These young doctors report high levels of anxiety, depression, and burnout. The question is: “What causes mental health issues among medical professionals?” Due to the multifactorial nature of anxiety, depression, and other psychiatric illnesses, there is no simple answer.

Young doctors must make a great effort to adapt to professional and personal changes during their 1st year of residency. This is a critical year in medical education because residents are expected to gain more autonomy in clinical decisions, greater knowledge of evidence-based treatment, and begin their role as a supervisors of medical students.^5^ Although 1st-year residents still have fewer responsibilities than senior residents, they are at the bottom of the hierarchy. During the 1st year, the resident goes back to the lowest level of the hierarchy, the youngest of the doctors, which is a vulnerable position.^6^,^7^

In the 1960s, Pierre Bourdieu^8^ discussed how the power structures of society are replicated in the education system. Those possessing “symbolic capital” dominate those who do not. Such practices, or *habitus* in Bourdieu’s terminology, are reproduced everywhere, including the academic environment, perpetuating dominant ideas until they become accepted without question. This perfect mechanism for maintaining the *status quo* is analogous to the concept of hidden curriculum in academia, which has been defined as: “learning that occurs through informal interactions among students, faculty, and others and/or learning that occurs through organizational, structural, and cultural influences intrinsic to training institutions.”^9^

In medical residency, knowledge and social connections are the symbolic capital that residents strive after to guarantee recognition.^10^ In this system, moral harassment is a *habitus*. Hirigoyen^11^ defined moral harassment as any abusive verbal or non-verbal conduct that, through repetition or systematization, causes humiliation, distress, or physical harm to a person. Since this definition emphasizes that harassment is a moral issue, it can only be characterized within a code of conduct defined by society or groups within society and accepted by each individual. What is perceived as harassment may vary from one individual or one social group to another over time. It tends to occur more frequently in rigid and bureaucratic organizations involving work overload and hierarchies,^12^,^13^ such as the medical environment. Aggressive behavior may be more tolerated in some scenarios, which might lead individuals not to recognize harassment when it occurs.^14^

Various authors have hypothesized that moral harassment during medical training is related to suboptimal learning experiences and to social, behavioral, emotional, psychological, and physical distress.^15^-^21^ Nevertheless, to our knowledge, no longitudinal study has investigated the causes of stress among medical residents. The present study attempts to identify whether moral harassment is a cause of anxiety, depression, and burnout among medical residents.

## Methods

We conducted a longitudinal study to assess moral harassment experiences among medical residents during medical school and their 1st year of residency.

### Procedures

All 1st-year residents at a tertiary university hospital in São Paulo, SP, Brazil in 2017 were contacted after enrolment. The residents initially received a paper-and-pencil version of the questionnaire during enrolment or the introductory course (1 or 2 weeks before the residency program started). Due to the logistical difficulties of contacting the residents and receiving the questionnaires once the residency began, we decided to provide the questionnaire in an online format. After enrolment, the first author sent periodic messages to the residents. At this point she was a peer, as she was a 2nd-year psychiatry resident. Throughout the process, she took great care to reinforce that the study aimed to give voice to the residents.

The residents were contacted during three stages: stage 1 – between enrolment and during the 1st month; stage 2 – after 6 months of residency; stage 3 – during the last month of their 1st year of residency. Of the 218 1st-year residents who were recruited, 76 (34.9%) agreed to participate in all study stages.

We advised the residents to answer the paper-and-pencil questionnaires when higher-ranking residents, professors, and other professionals were not present. Online questionnaires could be answered at home, after training. To guarantee confidentiality throughout the study, residents received a questionnaire access number linked to their e-mail, enabling us to send reminder e-mails to maximize adhesion.

### Measures

Stage 1 consisted of four questionnaires: 1) general demographics (personal, academic, and professional data, except the participant’s name to ensure confidentiality); 2) the Patient Health Questionnaire-4; 3) the Maslach Burnout Inventory Human Services Survey; 4) questions about harassment during medical school. Stages 2 and 3 consisted of three questionnaires: 1) the Patient Health Questionnaire-4; 2) the Maslach Burnout Inventory Human Services Survey; and 3) questions on harassment during the previous 6 months of residency. We decided to ask about harassment during medical school in stage 1 to compare changes in perceived harassment from the beginning of the residency to the middle and the end of the 1st year.

The Patient Health Questionnaire-4 is a self-report questionnaire consisting of four questions scored on a Likert-type scale that assesses depression and anxiety symptoms: the higher the score, the higher the symptom intensity. We used the Brazilian Portuguese version of the instrument, which was translated by Pfizer^22^ and has been previously validated.^23^,^24^

The Maslach Burnout Inventory Human Services Survey is a self-report questionnaire designed for professionals in human services (nurses, physicians, health aides, social workers, health counselors, therapists, etc.), that addresses three dimensions: emotional exhaustion (a lack of emotional energy to cope with work tasks); depersonalization (a coping mechanism in which individuals treat themselves and others with indifference or affective distance); and reduced personal accomplishment (a negative evaluation of one’s capabilities to perform the job effectively and discontent towards one’s profession). It has been validated in Brazilian Portuguese.^25^

The harassment questions were based on a Brazilian questionnaire used to assess harassment among medical students^26^ and a similar questionnaire used in a Japanese study to assess harassment and abuse during medical residency.^27^ We asked the residents whether they were harassed by anyone in an academic setting (fellow residents, patients, supervisors, or other health professionals), following the classifications suggested by Hirigoyen (1998/2003)^28^: 1) social isolation or refusal to communicate; 2) violation of dignity (humiliation/depreciation related to ethnic, religious, political, or other characteristics); 3) abuse of authority regarding work or academics (forced to perform unethical tasks, threats of condemnation, etc.); and 4) verbal (shouting), physical (threats, kicking, punching), or sexual (assault or discrimination) harassment. For each type of harassment, we inquired about the disturbance it caused in the victim, as described in another study.^29^

The question: “How much did this type of harassment disturb you?” had a multiple-choice answer: none, a little, and a lot. The answers “a little” and “a lot” were grouped during statistical analysis, since there was no significant difference between them, resulting in a dichotomous variable (disturbed or not disturbed). Thus, participants could recognize that they suffered moral harassment, by definition, but did not consider it disturbing, possibly accepting the violence.

The final question “How did you react after suffering harassment?” had a multiple-choice answer: Nothing, because it is just a phase and will end soon; Nothing, for fear of retaliation; I confronted the aggressor directly; I registered a complaint with the ethics committee; I sought psychological support; I spoke to colleagues, family, or friends, who suggested I do nothing. To enable statistical analysis, these answers were grouped into three categories:

1. Did nothing: “Nothing, because it is just a phase and will end soon”; “Nothing, for fear of retaliation.”
2. Confronted the aggressor: “I confronted the aggressor directly”; “I registered a complaint with the ethics committee.”
3. Asked for support: “I sought psychological support”; “I spoke to colleagues, family, or friends, who suggested I do nothing.”

For the statistical analysis, the residency programs were grouped into three categories: (a) clinical specialties (acupuncture, dermatology, emergency medicine, family medicine, internal medicine, neurology, physical and rehabilitation medicine, psychiatry, sports medicine, and traffic medicine); (b) surgical specialties (general surgery, neurosurgery, obstetrics, and gynecology, ophthalmology, orthopedics and traumatology, and otorhinolaryngology; and (c) diagnostic and therapeutic support (anesthesiology, clinical pathology, laboratory pathology, medical genetics, radiology, and radiotherapy).

To test comprehension of each question, a pilot study was conducted with six 5th-year medical students 3 months before the actual study began. No questions required reformulation, and these responses were not used in the final analysis.

### Statistical analysis

Initially, descriptive statistics were used (frequency and relative frequency distribution, mean, and SD). Pearson’s chi-square analysis or Fischer’s test (for small samples) was used to test for statistical differences in proportions or the distribution of nominal data. The Kruskal-Wallis test was used to compare means between three or more groups. Once the mean differences were detected through the Kruskal-Wallis test, the differences were defined through the Dunn-Bonferroni test, with p < 0.01 considered statistically significant. In logistic regression, mental health outcomes were considered dependent variables and harassment was a predictor variable.

To investigate the effects of moral harassment during the 1st year of residency, participants were classified into two groups: group 1 did not suffer harassment during the 1st or 2nd semester; group 2 suffered harassment during the 1st and/or 2nd semester. Control variables included sex, age, and medical specialty.

The analysis was performed using SPSS and STATA.

### Ethics statement

This project was approved by the Universidade Federal de São Paulo research ethics committee (decision 65511117.3.1001.5505).

## Results

### Demographics

A total of 192 residents participated in this study, and 76 (39.58%) answered the questionnaire in all three stages. Table 1 describes the main characteristics of this group.

### Mental health measures

**Figure 1:**
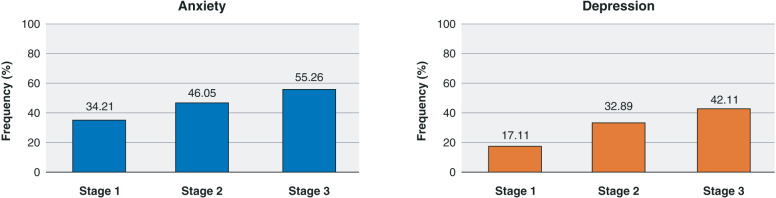
The participants’ anxiety and depression levels during each study stage.

Categorical analysis indicated statistically significant variations between stages for anxiety symptoms (p = 0.033) and depression symptoms (p = 0.003) (Figure 1). The Dunn and Friedman tests indicated the same for anxiety and depression scores (Supplementary Table S1).

**Figure 2:**
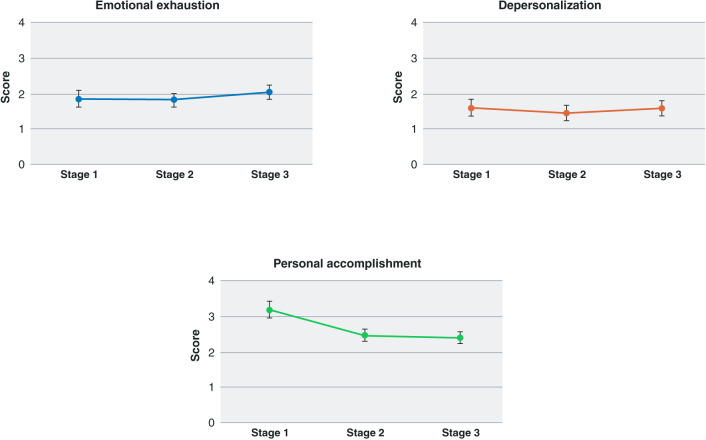
Burnout dimension scores during each study stage.

The Friedman test indicated significant variation in burnout (p < 0.001), although there was no significant variation in the emotional exhaustion (p = 0.3579) or depersonalization (p = 0.0685) dimensions. The Dunn test indicated that personal realization scores were significantly higher in stage 1 than in stage 2 (p < 0.0001) or stage 3 (p < 0.0001) (Table 2 and Figure 2).

### Harassment measures

**Figure 3:**
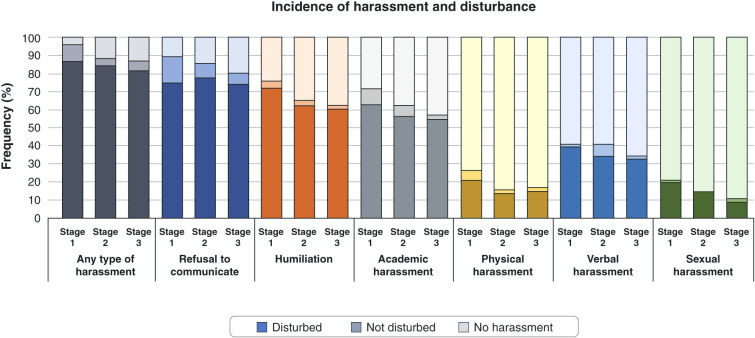
Harassment and disturbance due to harassment in each study stage according to type.

The occurrence of harassment was high (> 90% of the residents) throughout the 1st year of residency, especially the refusal to communicate and humiliation types. For almost every type of harassment, a small number of residents reported not being disturbed by it. The only exception was sexual harassment during the 1st semester, for which all victims reported disturbing (Figure 3).

In general, the frequency of harassment decreased over time. However, no significant chi-square differences were found among individual types of harassment or groups (Figure 3 and Supplementary Table S2). Although disturbance tended to be greater in stage 3, no significant differences were observed among harassment types or groups (Figure 3 and Supplementary Table S2).

### Causes of mental health problems during the first year of residency

Initially, logistic regression was used to determine whether sex, age, medical specialty, and having suffered any harassment affected mental health. In the multivariate analysis (Table 3), depression scores and emotional exhaustion scores were 1.10 and 0.58 points lower among surgical residents, indicating a smaller increase than among clinical residents.

We ran a model including the victims’ response to harassment. As shown in Table 4, residents who suffered harassment and confronted the aggressor had a 1.88 point larger variation in anxiety than victims who did nothing (coefficient = 1.877; 95%CI 0.686-3.068; p = 0.002). When analyzing depression as an outcome, the surgical residents’ score was 1.06 points lower than clinical residents (coefficient = -1.056; 95%CI -2.008 to -0.104; p = 0.030) in the multivariate analysis (Table 4).

In the multivariate analysis of burnout dimensions as outcomes, surgical residents had lower emotional exhaustion scores than clinical residents (coefficient = -0.545; 95%CI -1.069 to -0.022; p = 0.041). Moreover, residents who suffered harassment and sought help had a 0.71 point larger variation in emotional exhaustion than those who suffered harassment and did nothing (coefficient = 0.712; 95%CI 0.110-1.313; p = 0.020).

Finally, residents who confronted their aggressors had a 0.75 point larger variation in emotional exhaustion than those who suffered harassment but did nothing (coefficient = 0.753; 95%CI 0.174-1.332; p = 0.011). No co-variables were associated with depersonalization or personal accomplishment between stages 1 and 3.

## Discussion

In summary, our main findings were:

1. There was significant variation in anxiety and depression scores and their frequency during the 1st year of residency. In particular, there was a significant decrease in the personal accomplishment dimension of burnout during the 1st year of residency.
2. The occurrence of harassment was extremely high throughout the 1st year of residency, which tended to decrease over time, but not significantly so. Most victims were disturbed by the harassment, but the proportion of non-disturbed residents tended to decrease in stage 3, although this trend was not significant.
3. The regression analysis indicated that:
  - Harassment victims who confronted their aggressors had more anxiety and emotional exhaustion than those who did nothing.
  - Residents who sought help had more emotional exhaustion than those who did nothing.
  - Surgical residents had a smaller increase in depression and emotional exhaustion than clinical residents.

Parro-Pires et al.^30^ conducted a longitudinal study of 111 Brazilian medical residents, finding increased depressive symptoms throughout the 1st year of residency, with a 9.01% incidence of depressive symptoms. This value is much smaller than that of the present study (17.11%-42.11%). It is also smaller than that found by Mata et al.^31^ in a systematic review (28.8%, range 20.9-43.2%), in which it increased during the calendar year. In another Brazilian study, Peterlini et al.^32^ found no association between the year of residency and depressive symptoms, unlike the results of the 2018 study and our study. Peterlini et al.^32^ also studied anxiety among residents and found no significant variations in state or trait anxiety between the 1st and 2nd semesters of residency, although we did.

We found two previous studies that longitudinally assessed burnout among residents^33^,^34^: one involving pediatric residents and another with family medicine residents. Both studies found an increase in emotional exhaustion and depersonalization during the 1st months of residency, while personal accomplishment decreased among pediatric residents, as found in our study. Several points must be made about burnout:

1. The expression of depersonalization may vary among different cultures and it may even vary among different medical specialties.^35^
2. Personal accomplishment may rapidly decrease during the 1st months of residency due to “normalization” of the resident role, which may have been over-idealized, as has been observed in medical students.^36^
3. The three burnout dimensions do not always change simultaneously and are not correlated enough to be considered a one-dimensional phenomenon,^37^ as some studies have tried to demonstrate.^38^ Therefore emotional exhaustion itself might not be enough to characterize burnout. This is why the most recent burnout studies have suggested using latent profiles instead of the three dimensions.^39^
4. Although the use of percentiles and cut-off points has been questioned in the literature,^39^ we decided to use them to be able to compare our results with other studies.
5. There is a discussion about whether burnout (especially emotional exhaustion) and depression could be considered the same variable.^40^ In our study, these measures did not parallel one another. Are burnout and depression two different perceived phenomena? Is depression more related to personal than work-related issues? Because medical residency could be seen as both a “state of being” and “where one lives,” the definition and analysis of burnout in this population may differ from that used for the general population. Since medical residents do not have the typical labor issues or interpersonal relations of blue- or white-collar workers, how is burnout to be assessed in this population?

The varying mental health results in different studies may be due to sample size and specificity (a single institution or residency program), and larger studies may help determine whether mental health variations are related to the passing of time. Nevertheless, the alarming amount of mental suffering among 1st-year medical residents should not be minimized, and interventions during the 1st year of residency are necessary. As can be seen below, these points may be better addressed through qualitative research.

In our study, 88.2 and 86.8% of the residents reported having been victims of some type of harassment during the 1st and 2nd semesters, respectively. These numbers are higher than the pooled prevalence found in two metanalyses (63.4 and 64.1%).^41^,^42^ Although we did not find significant variation in the occurrence of harassment and disturbance due to harassment between stages, it is important to note that from a sociological perspective, the observed variation could be related to three factors:

1. As residents go through their 1st year, they gradually ascend in the hierarchical structure and, hence, may be less exposed to harassment.
2. As residents progress towards the 2nd year and ascend in the hierarchical structure, they may become less tolerant of abusive acts.
3. As residents progress, they may also incorporate the *habitus*, accepting these behaviors (being initiated into the hidden curriculum) and thus acknowledging fewer attitudes as abusive, considering them “part of their education.”^10^

Our main study question was “Does moral harassment cause anxiety, depression, and burnout in medical residents?” This has been hypothesized in other studies,^42^,^43^ but prospective studies have not established causality. Since self-perceived mental health status is multicausal, it was not surprising that we did not find a direct relationship between harassment and lower mental health.

According to our findings, confronting one’s antagonist or seeking help led to greater mental suffering. It may be challenging to change medical education or confront harassment, given that the results indicate that residency programs are not safe environments for such initiatives due to their hierarchical structure and violence.

The fact that 1st-year surgical residents reported less depressive and anxious symptoms is curious. Often, surgical residency programs tend to be more hierarchical and traditional, and one might expect this to lead to more severe mental problems. Specific in-depth research concerning these questions, including analysis of sex differences, could clarify whether surgical residents are more resilient, have less awareness, or are more committed to the hidden curriculum, accepting its structure and, consequently, suffering less. A recent systematic review^44^ indicated that the majority of surgeons in training who have been harassment victims do not report it for fear of retaliation. In this case, silence would be a defense mechanism to avoid greater mental distress. Based on these results, it is evident that institutional measures are needed to create an environment in which those disturbed by harassment can express themselves and demand change.

To the best of our knowledge, this is the first longitudinal study to assess mental health and harassment among medical residents. Although we have raised more questions than answers, we hope our results lead to greater reflection and debate. It should be noted that because harassment is a moral issue, perception of it will vary among social groups, individuals, and historical periods. The medical community is not immune to these changes, and traditional structures should be reexamined to create a healthy environment for all professionals. Discourse about curriculum change, new ways to evaluate students, and modern educational techniques is of no avail if harmful structural aspects of medical culture and interpersonal relationships persist. Institutional changes to the *habitus* are required to create a place where residents feel they can express themselves.

## Disclosure

The authors report no conflicts of interest.

## Figures and Tables

**Table 1:** Sociodemographic data of 76 1st-year residents from a public university hospital in São Paulo, Brazil, 2017

Characteristics	
Sex	
Female	35 (46.05)
Male	41 (53.95)
Age	
< 25	21 (27.63)
26 to 30	48 (63.16)
≥ 30	7 (9.21)
Sexuality	
Heterosexual	71 (93.42)
Bisexual	3 (3.95)
Homosexual	2 (2.63)
Type of medical university	
Private	9 (11.84)
Public	67 (88.16)
Medical residency specialty	
Clinical	44 (57.89)
Surgical	18 (23.68)
DTS	14 (18.42)
Total	76 (100.00)

Data presented as n (%).

DTS = diagnostic and therapeutic support.

**Table 2:** Distribution of 76 1st-year residents by burnout dimensions during each study stage

Dimensions	Stage 1	Stage 2	Stage 3
Emotional exhaustion	1.87 ± 1.04 (1.67)	1.82 ± 0.89 (1.67)	2.04 ± 0.90 (2.06)
Depersonalization	1.60 ± 1.01 (1.40)	1.44 ± 0.85 (1.40)	1.58 ± 0.88 (1.60)
Personal accomplishment	3.19 ± 0.84 (3.31)	2.47 ± 0.66 (2.37)	2.39 ± 0.69 (2.50)

Data presented as mean ± SD (median).

**Table 3:** Logistic regression for anxiety, depression, and burnout dimensions (emotional exhaustion, depersonalization and personal accomplishment) between stages 1 and 3 based on harassment victimization

	Anxiety			Depression			Emotional exhaustion			Depersonalization			Personal accomplishment		
	Univariate analysis	Multivariate analysis		Univariate analysis	Multivariate analysis		Univariate analysis	Multivariate analysis		Univariate analysis	Multivariate analysis		Univariate analysis	Multivariate analysis	
	Coefficient (95%CI)	Coefficient (95%CI)	p-value	Coefficient (95%CI)	Coefficient (95%CI)	p-value	Coefficient (95%CI)	Coefficient (95%CI)	p-value	Coefficient (95%CI)	Coefficient (95%CI)	p-value	Coefficient (95%CI)	Coefficient (95%CI)	p-value
Sex															
Female	Ref.	Ref.	-	Ref.	Ref.	-	Ref.	Ref.	-	Ref.	Ref.	-	Ref.	Ref.	-
Male	-0.195	-0.212 (-1.164 to 0.740)	0.658	-0.274	-0.342 (-1.138 to 0.454)	0.394	-0.021	-0.073 (-0.534 to 0.389)	0.755	0.061	0.014 (-0.464 to 0.492)	0.954	0.106	0.192 (-0.208 to 0.592)	0.342
Age	-0.037	-0.041 (-0.136 to 0.055)	0.401	-0.045	-0.057 (-0.137 to 0.023)	0.158	0.009	0.006 (-0.041 to 0.052)	0.805	-0.004	-0.010 (-0.059 to 0.038)	0.667	-0.027	-0.021 (-0.062 to 0.019)	0.292
Medical specialty															
Clinical	Ref.	Ref.	-	Ref.	Ref.	-	Ref.	Ref.	-	Ref.	Ref.	-	Ref.	Ref.	-
Surgical	-0.747	-0.845 (-2.003 to 0.312)	0.150	-0.957	-1.102 (-2.070 to -0.134)	**0.026**	-0.578	-0.585 (-1.142 to -0.028)	**0.040**	-0.290	-0.300 (-0.877 to 0.277)	0.303	0.038	0.014 (-0.468 to 0.497)	0.953
DTS	-0.351	0.030 (-1.646 to 1.705)	0.972	-0.639	-1.030 (-2.432 to 0.371)	0.147	-0.450	-0.300 (-1.111 to 0.511)	0.463	-0.517	-0.648 (-1.488 to 0.191)	0.128	0.151	0.540 (-0.163 to 1.242)	0.130
Harassment in stages 2 and/or 3															
No	Ref.	Ref.	-	Ref.	Ref.	-	Ref.	Ref.	-	Ref.	Ref.	-	Ref.	Ref.	-
Yes	0.671	0.865 (-1.329 to 3.060)	0.434	-0.625	0.089 (-1.296 to 1.474)	0.898	0.357	0.276 (-0.687 to 1.239)	0.570	0.231	-0.226 (-1.223 to 0.771)	0.652	0.298	0.708 (-0.126 to 1.543)	0.095

DTS = diagnostic and therapeutic support.

Bold type denotes statistical significance.

**Table 4:** Logistic regression for anxiety, depression, and burnout dimensions (emotional exhaustion, depersonalization, and personal accomplishment) between stages 1 and 3 according to harassment response

	Anxiety			Depression			Emotional exhaustion			Depersonalization			Personal accomplishment		
	Univariate analysis	Multivariate analysis		Univariate analysis	Multivariate analysis		Univariate analysis	Multivariate analysis		Univariate analysis	Multivariate analysis		Univariate analysis	Multivariate analysis	
	Coefficient (95%CI)	Coefficient (95%CI)	p-value	Coefficient (95%CI)	Coefficient (95%CI)	p-value	Coefficient (95%CI)	Coefficient (95%CI)	p-value	Coefficient (95%CI)	Coefficient (95%CI)	p-value	Coefficient (95%CI)	Coefficient (95%CI)	p-value
Sex															
Female	Ref.	Ref.	-	Ref.	Ref.	-	Ref.	Ref.	-	Ref.	Ref.	-	Ref.	Ref.	-
Male	-0.195	0.128 (-0.806 to 1.061)	0.789	-0.274	-0.226 (-1.051 to 0.600)	0.592	-0.021	0.105 (-0.349 to 0.559)	0.650	0.061	0.060 (-0.436 to 0.555)	0.813	0.106	0.222 (-0.191 to 0.635)	0.292
Age	-0.037	-0.063 (-0.155 to 0.028)	0.176	-0.045	-0.065 (-0.146 to 0.016)	0.117	0.009	-0.010 (-0.054 to 0.035)	0.672	-0.004	-0.012 (-0.061 to 0.037)	0.627	-0.027	-0.030 (-0.070 to 0.011)	0.152
Medical residency specialty															
Clinical	Ref.	Ref.	-	Ref.	Ref.	-	Ref.	Ref.	-	Ref.	Ref.	-	Ref.	Ref.	-
Surgical	-0.747	-0.740 (-1.817 to 0.336)	0.178	-0.957	-1.056 (-2.008 to -0.104)	**0.030**	-0.578	-0.545 (-1.069 to -0.022)	**0.041**	-0.290	-0.283 (-0.854 to 0.288)	0.332	0.038	0.007 (-0.470 to 0.483)	0.978
DTS	-0.351	0.100 (-1.459 to 1.660)	0.900	-0.639	-0.991 (-2.370 to 0.389)	0.159	-0.450	-0.368 (-1.126 to 0.390)	0.342	-0.517	-0.656 (-1.484 to 0.171)	0.120	0.151	0.481 (-0.210 to 1.171)	0.172
Occurrence/response after harassment															
Suffered harassment and did nothing	Ref.	Ref.	-	Ref.	Ref.	-	Ref.	Ref.	-	Ref.	Ref.	-	Ref.	Ref.	-
Suffered harassment and sought help	0.958	1.167 (-0.071 to 2.404)	0.065	0.267	0.377 (-0.717 to 1.472)	0.499	0.638	0.712 (0.110 to 1.313)	**0.020**	0.035	0.110 (-0.547 to 0.767)	0.744	0.138	0.287 (-0.261 to 0.835)	0.305
Suffered harassment and confronted the aggressor	1.839	1.877 (0.686 to 3.068)	**0.002**	0.857	0.793 (-0.260 to 1.846)	0.140	0.772	0.753 (0.174 to 1.332)	**0.011**	0.346	0.331 (-0.301a 0.963)	0.305	-0.235	-0.159 (-0.686 to 0.368)	0.553
Did not suffer harassment	-0.089	-0.254 (-2.339 to 1.832)	0.812	0.143	0.849 (-0.996 to 2.693)	0.367	-0.046	0.162 (-0.852 to 1.176)	0.754	-0.154	0.387 (-0.720 to 1.494)	0.493	-0.324	-0.682 (-1.605 to 0.241)	0.148

DTS = diagnostic and therapeutic support.

Bold type denotes statistical significance.
