# Supplementary material for: Moral harassment and mental health in medical residents: a longitudinal study
Source: Braz J Psychiatry. 2025 Jan 22;47:e20243579. doi: 10.47626/1516-4446-2024-3579 (PMC12679693; doi:10.47626/1516-4446-2024-3579)
Supplement: Supplementary file 1 [file bjp-47-e20243579-suppl1.pdf]

http://doi.org/10.47626/1516-4446-2024-3579

Supplementary Table S1 Patient Health Questionnaire-4 (PHQ) scores for anxiety and depression in the 3 stages of the study

	**Stage 1**	**Stage 2**	**Stage 3**
Anxiety			
PHQ1	1.38±0.89 (1)	1.63±0.92 (1)	1.71±0.95 (2)
PHQ2	0.67±0.82 (0)	1.05±1.02 (1)	1.24±1.04 (1)
Total	2.05±1.56 (2)	2.68±1.82 (2)	2.95±1.87 (3)
Depression			
PHQ3	0.75±0.83 (1)	1.21±0.83 (1)	1.37±0.91 (1)
PHQ4	0.55±0.82 (0)	0.95±0.91 (1)	1.16±0.98 (1)
Total	1.30±1.52 (1)	2.16±1.55 (2)	2.53±1.80 (2)

Data presented as mean ± SD (median).
The Friedman test indicates significant variations over time for total anxiety (p = 0.0005) and total depression (p < 0.0001). The same results were observed the Dunn test (significantly lower total anxiety and total depression scores in Stage 1 than Stage 2 (p = 0.0177 vs p = 0.0002, respectively) and Stage 3 (p=0.0010 vs p=0.0001, respectively). The variations in anxiety and depression scores between Stages 1 and 3 were, respectively, 0.89±2.02 and 1.22±1.74 (positive values indicate higher scores in Stage 3 than to Stage 1).

Supplementary Table S2 Occurrence of harassment/disturbance in the 3 stages of the study

	**Stage 1**	**Stage 2**	**Stage 3**
Social isolation			
Disturbed	57 (75.00)	59 (77.63)	56 (73.68)
Not disturbed	11 (14.47)	6 (7.89)	5 (6.58)
No harassment	8 (10.53)	11 (14.47)	15 (19.74)
Violation of dignity			
Disturbed	55 (72.37)	47 (61.84)	46 (60.53)
Not disturbed	3 (3.95)	3 (3.95)	1 (1.32)
No harassment	18 (23.68)	26 (34.21)	29 (38.16)
Work/academic harassment			
Disturbed	48 (63.16)	43 (56.58)	42 (55.26)
Not disturbed	6 (7.89)	4 (5.26)	1 (1.32)
No harassment	22 (28.95)	29 (38.16)	33 (43.42)
Physical harassment			
Disturbed	16 (21.05)	11 (14.47)	11 (14.47)
Not disturbed	4 (5.26)	1 (1.32)	2 (2.63)
No harassment	56 (73.68)	64 (84.21)	63 (82.89)
Verbal harassment			
Disturbed	30 (39.47)	26 (34.21)	25 (32.89)
Not disturbed	1 (1.32)	5 (6.58)	1 (1.32)
No harassment	45 (59.21)	45 (59.21)	50 (65.79)
Sexual harassment			
Disturbed	15 (19.74)	11 (14.47)	7 (9.21)
Not disturbed	1 (1.32)	0 (0.00)	1 (1.32)
No harassment	60 (78.95)	65 (85.53)	68 (89.47)
Any harassment			
Disturbed	**66 (86.84)**	**64 (84.21)**	**62 (81.58)**
Not disturbed	**7 (9.21)**	**3 (3.95)**	**4 (5.26)**
No harassment	**3 (3.95)**	**9 (11.84)**	**10 (13.16)**

Data presented as n (%).
